# Structure and Spatial Heterogeneity of Chemosynthesis‐Based Deep‐Sea Archaeal and Bacterial Communities in Western South Atlantic

**DOI:** 10.1002/ece3.72973

**Published:** 2026-02-17

**Authors:** Taiz L. Lopes Simão, Karine A. Felix Ribeiro, Raquel Dias, Adolpho H. Augustin, Luiz F. Rodrigues, Dennis J. Miller, Adriano R. Viana, Eric W. Triplett, João M. M. Ketzer, Adriana Giongo, Eduardo Eizirik, Renata Medina‐Silva

**Affiliations:** ^1^ Pontifical Catholic University of Rio Grande do Sul School of Health and Life Sciences Porto Alegre RS Brazil; ^2^ Department of Microbiology and Cell Science University of Florida Gainesville Florida USA; ^3^ Pontifical Catholic University of Rio Grande do Sul Institute of Petroleum and Natural Resources Porto Alegre RS Brazil; ^4^ Petrobras Centro de Pesquisas e Desenvolvimento Leopoldo Américo Miguez de Mello (CENPES) Rio de Janeiro RJ Brazil; ^5^ Petrobras EandP‐EXP Rio de Janeiro RJ Brazil

**Keywords:** chemosynthetic communities, deep‐sea microbiome, metabarcoding, methane seep, sulfate–methane transition zone

## Abstract

Cold seeps are widespread deep‐sea ecosystems sustained by methane‐rich fluid seepage and host dense chemosynthesis‐based biological communities. In 2016, a methane‐driven chemosynthetic system was discovered on the Rio Grande Cone, in the Western South Atlantic Ocean, but the structure and drivers of its prokaryotic communities remained poorly understood. Here, we investigated archaeal and bacterial communities associated with deep‐sea sediments across three geographic areas (A, C, and E) and a vertical gradient of up to 18 m below the seafloor, encompassing sediment layers within and below the sulfate–methane transition zone (SMTZ). Community composition was assessed using high‐throughput sequencing of the 16S rRNA gene (V3‐V4 region), processed into amplicon sequence variants (ASVs), and related to local geochemical gradients using multivariate analyses. To disentangle the ecological responses of methane‐cycling taxa from the broader microbiome, the prokaryotic community was analyzed by contrasting the ANME‐SRB consortium with the remaining archaeal and bacterial taxa. Both groups exhibited significant spatial structuring across areas and sediment layers. Methane concentration and depth were the dominant drivers shaping both ANME‐SRB and the remaining prokaryotic community, with conductivity further influencing the latter. Core microbiome analysis revealed a small number of widespread taxa accounting for a large proportion of total community abundance, including an atypical dominance of the archaeal genus *Sulfophobococcus*. Functional predictions indicated a predominance of sulfur‐ and nitrogen‐related metabolisms, with no clear depth‐structured metabolic profiles across the SMTZ. Overall, our results highlight how local geochemical gradients shape both methane‐cycling and non‐methane‐cycling prokaryotic assemblages in a poorly explored South Atlantic cold seep, providing a baseline for future genome‐resolved investigations of microbial functioning in this system.

## Graphical abstract

We report an extensive investigation of prokaryotic communities from a methane cold‐seep area in the Western South Atlantic Ocean (Southern Brazil) employing a broad sample set, including three distinct geographic areas (A, C and E) across a depth gradient (up to 18 m below the seafloor) related to the sulfate–methane transition zone (SMTZ). Results indicated differences in community composition among the surveyed areas, and from shallow to deeper sediment layers related to the SMTZ, which were mainly influenced by the methane gradients. Functional predictions revealed that bacterial and archaeal communities are predominantly involved in sulfur and nitrogen cycles, although no depth‐structured metabolic profiles were detected, revealing undescribed roles of microbial communities occurring in this poorly explored area, contributing to a deeper understanding of cold seeps' microbiota diversity and functioning.

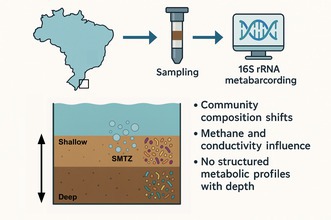

## 1 Introduction

Deep‐sea sediments represent some of the richest pools of biota on Earth (Guardiola et al. 2015), extending for kilometers below the seafloor (Ciobanu et al. 2014; Inagaki et al. 2015). Cold seeps, or methane seeps, are island‐like ecosystems in deep‐sea sediments characterized by low temperatures (2°C–4°C) and seafloor seepage of methane and other hydrocarbons from seabed reservoirs (Arvidson et al. 2004), harboring a rich and abundant biological community supported by chemosynthesis (Boetius and Wenzhöfer 2013). These communities exhibit high biomass and endemicity, highly adapted organisms compared to surrounding areas, unique symbiotic relationships between bacteria and macrofauna, as well as some taxa that are shared among cold seeps and other deep‐sea reducing sites, such as hydrothermal vents and organic falls (such as whales and wood falls) (Ruff et al. 2015; Xiao et al. 2023). The prokaryotic community at cold seeps comprises abundant bacteria and archaea that occur worldwide, typically associated with a substantial diversity of rare relatives, which together make these ecosystems important hotspots of biodiversity in the deep ocean (Ruff et al. 2015).

An essential component of the chemosynthesis‐based communities at cold seeps is the consortium formed by anaerobic methanotrophic (ANME) archaea and sulfate‐reducing bacteria (SRB), which couple the anaerobic oxidation of methane (AOM) to sulfate reduction, a very prevalent process in cold seeps ecosystems (Boetius et al. 2000; Knittel and Boetius 2009). Estimates indicate that the ANME‐SRB metabolism consumes 75% of the methane that reaches the seafloor surface, providing a globally significant role as a methane sink and controlling the emissions of this important greenhouse gas to the atmosphere (Reeburgh 2007; Boetius and Wenzhöfer 2013). Phylogenetic analyses have led to the classification of four major ANME lineages—ANME‐1a/b (Orphan et al. 2002), ANME‐2a,b,c (Orphan, House, et al. 2001; Orphan et al. 2002), ANME‐2d (Haroon et al. 2013), and ANME‐3 (Lösekann et al. 2007). The SRB groups include the Seep‐SRB1 (Orphan, Hinrichs, et al. 2001) in the *Desulfobacteraceae*, Seep‐SRB2 (Kleindienst et al. 2012), and Seep‐DBB (Green‐Saxena et al. 2014) in the *Desulfobulbaceae*, and HotSeep‐1 cluster (Holler et al. 2011) in the Deltaproteobacteria.

The prokaryotic diversity at cold seeps is mostly attributed to local environmental features, whose impacts can be observed both at the macrodiversity (i.e., the measure of population diversity within a community) (Ruff et al. 2015) and microdiversity (i.e., the measure of genetic variation within a population) (Dong et al. 2023) levels. Previous findings have shown that sediment depth (Dong et al. 2020), water depth, and seafloor temperature (Ruff et al. 2015), as well as local geochemistry (Yanagawa et al. 2011; Niu et al. 2017), are the key environmental factors that influence the distribution of microorganisms associated with cold seeps. Among the geochemical variables, methane, sulfate, sulfide, nitrate, and dissolved oxygen are among the most important in shaping cold seep microbiomes (Ruff et al. 2015; Cruaud et al. 2017; Semler et al. 2022; Dong et al. 2023).

The impact of geochemical gradients has also been observed in members of the ANME and SRB groups with niche differentiation based on ecophysiological differences (Roalkvam et al. 2012; Green‐Saxena et al. 2014), which can influence not only the distribution of the archaeal and bacterial taxa but also the ANME‐SRB associations. Moreover, the spatial distribution of the aggregates ANME‐SRB within the subseafloor sediments is often associated with layers corresponding to the sulfate–methane transition zone (SMTZ) (Boetius et al. 2000), which is characterized by the horizon where sulfate and methane coexist. Therefore, this interface is expected to be a significant driver of archaeal and bacterial diversity patterns in cold seeps, as it is supposed to divide two distinct metabolisms, i.e., sulfate‐reduction and methanogenesis (Harrison et al. 2009). Other metabolic functions are also expected to exhibit depth‐related variation, such as the methane consumption by aerobic methane‐oxidizing bacteria (MOB), mainly from the order Methylococcales, which is oxygen dependent and, therefore, prevalent in the upper oxic sediment layers of cold seeps (Ruff 2020).

In recent decades, considerable effort has been devoted to understanding the archaeal and bacterial diversity at deep‐sea cold seeps. However, the wide extension of these areas and their remote location result in still limited knowledge of these environments. Recently, the existence of a chemosynthesis‐driven biotic community off the coast of southern Brazil was first reported (Giongo et al. 2016), in a location where high levels of methane and the presence of gas hydrates have been detected (Miller et al. 2015; Rodrigues et al. 2017). These findings indicated, for the first time, that cold seep ecosystems are present in the western South Atlantic Ocean, presenting similar features to those discovered in other oceanic sites. In this context, the goal of this study was to expand knowledge of the archaeal and bacterial community in this cold seep site by characterizing the sediment‐associated microbiome at different areas and depths (up to 18 m below sea floor), and its environmental drivers (local geochemical gradients). We used a comparative approach between the ANME‐SRB sub‐community and the remaining prokaryotic community to test, in our study area, the hypothesis that (i) the sediment layer (within and below sulfate–methane transition zone) is the major driver of prokaryotic composition; (ii) regarding the role of geochemical gradients, methane and sulfate are more decisive for ANME‐SRB when compared to the remaining prokaryotic community; (iii) distinct prokaryotic composition associated with different sediment layers are reflected in depth‐structured metabolic profiles.

## 2 Materials and Methods

### 2.1 Study Site and Sampling

**FIGURE 1:**
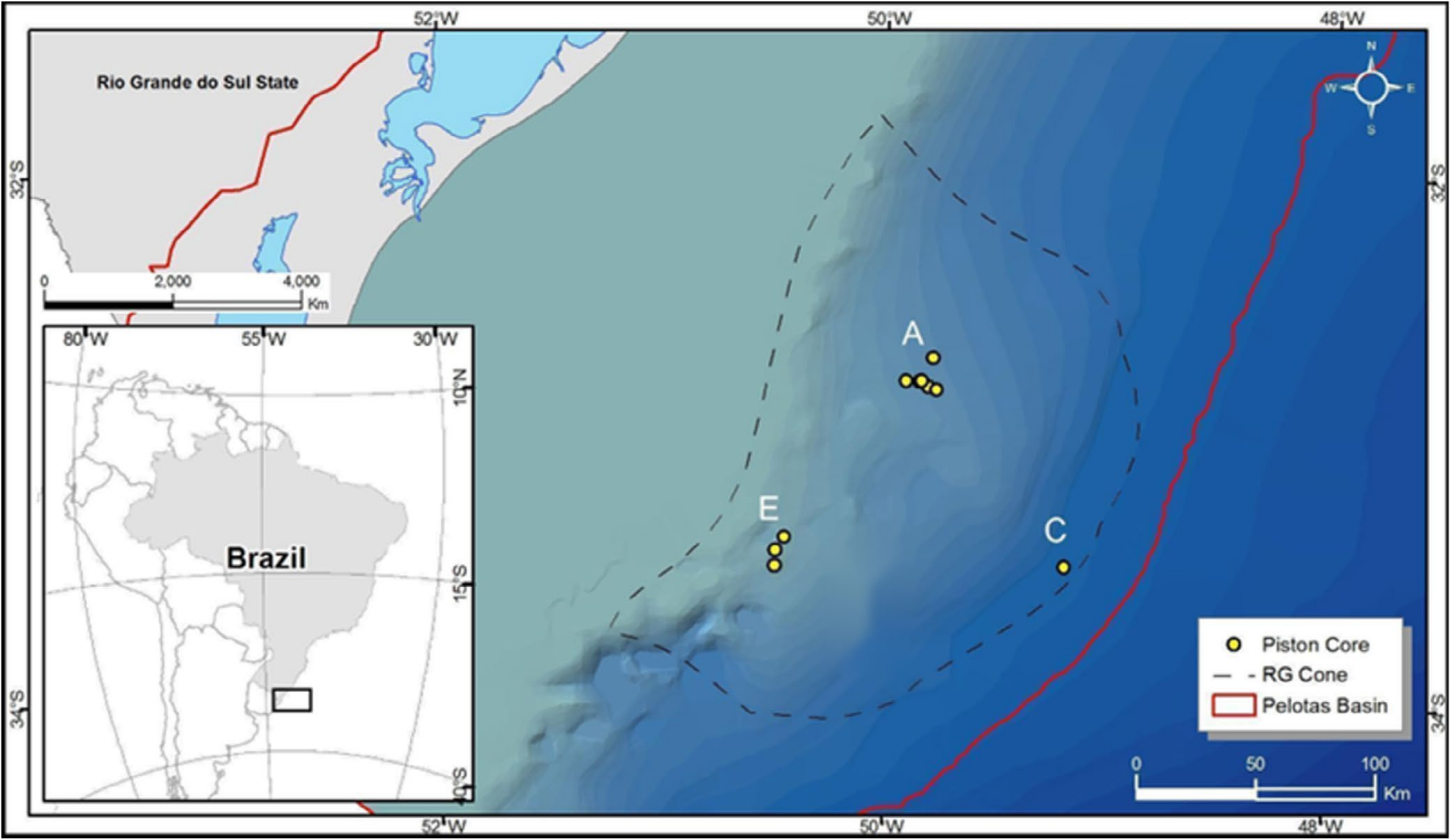
Site location. The Pelotas Basin is delimited by a red line; the Rio Grande Cone is bounded by a dashed line; the yellow dots represent the locations of piston cores sampled in this study, with letters indicating each of the surveyed areas (A, C, D, and E). Scale bar of 100 km is presented.

The study area is located in the northeastern flank of the Rio Grande Cone (Pelotas Basin), ca. 250 km off the southern Brazil coast (Figure 1). This area was previously characterized by high levels of methane, the presence of gas hydrates (Miller et al. 2015; Rodrigues et al. 2017) and of a chemosynthesis‐driven biotic community (Giongo et al. 2016). During the MD195 campaign aboard the French research vessel Marion Dufresne in July 2013, deep‐sea sediments were collected in four distinct areas through soil drilling using piston cores (Figure 1). The water column depth at the sampled sites ranged from 540 to 3052 m, and the average distance between areas was 50 to 100 km. Among these sediment samples, 59 were selected for this study, which were collected from seven piston cores (PC80, PC81, PC90, PC93, PC95, PC102, and PC109) in three different areas (A, C, and E). For each PC, sampling was performed with the following stratification: sediment surface, one‐meter spacing in the first three meters below seafloor (mbsf), i.e., 1, 2, and 3 mbsf, and three‐meter spacing down to 18 mbsf, i.e., 6, 9, 12, 15, and 18 mbsf. In some cases, the depth of the sediment sampled by the piston core did not reach the limit of 18 mbsf, so sampling was carried out to the deepest recovered layer. The collection routine was executed with sterile materials, and the sediment samples were immediately frozen at −80°C after sampling until DNA extraction, which was performed 4 months later.

### 2.2 Geochemical Characterization

Geochemical variables (chloride, sulfate, CO_2_, CH_4_, pH, and conductivity) in interstitial water and surrounding sediment were measured as described by Rodrigues et al. (2017) and are described in Appendix 1: Table A1. Considering that the methane concentration never reached *zero*, and the SMTZ is characterized by the presence of both compounds (methane and sulfate), the samples were classified into two categories: within and below SMTZ (Rodrigues et al. 2017). The lower limit of the SMTZ (i.e., the maximum depth where sulfate was detected) varied substantially among piston cores and was verified from the first centimeters below the seafloor until depths greater than 16 mbsf (Appendix 1: Table A1).

### 2.3 DNA Extraction, Amplification and Sequencing

Genomic DNA was extracted from three aliquots of 500 mg sediment according to the protocol described by Zhou et al. (1996), adding steps of freezing in liquid nitrogen and thawing at 70°C in a water bath three times to increase cell lysis efficiency. The extracted DNA from each aliquot of a sample was pooled together and purified with the QIAquick PCR Purification Kit (QIAGEN). Fragments of the 16S rRNA gene were amplified using the universal primers 515F and 806R (Bates et al. 2011), which amplify 291 bp from the V3‐V4 hypervariable region of this prokaryotic gene. The thermocycling conditions were performed as previously described (Medina‐Silva et al. 2018), except for the initial denaturation time, modified to 4 min instead of 45 s. The PCR amplicon purification and high throughput sequencing were executed as described by Medina‐Silva et al. (2018).

### 2.4 Bioinformatic Analyses

Sequences from the 16S rRNA gene were preprocessed and classified using the DADA2 (Divisive Amplicon Denoising Algorithm) pipeline (v. 1.16) (Callahan et al. 2016) in R (v. 4.3.3) (R Core Team 2021). After inspecting read quality profiles, quality‐trimming and filtering were performed using the *FilterAndTrimmed* function with the following parameters: truncQ = 2, maxN = 0, maxEE = 2, and minLen = 100. Error rates were inferred from the filtered reads using the *learnErrors* function, and ASVs were inferred using the sample‐specific error model. Chimeric sequences were identified and removed using the removeBimeraDenovo function with the consensus method, retaining only high‐confidence non‐chimeric ASVs. Reads shorter than 100 bp were removed, and 2 expected errors per read were allowed. The subsequent steps included error inference, denoising, and chimera removal (Appendix 2: Table A2). The returned amplicon sequencing variants (ASVs) were taxonomically assigned using the SILVA database (v. 138) (Quast et al. 2012) and imported into the phyloseq R package for subsequent analyses (McMurdie and Holmes 2013). The taxonomic nomenclature was maintained as returned by the SILVA database.

### 2.5 Archaeal and Bacterial Community Analyses

**FIGURE A1:**
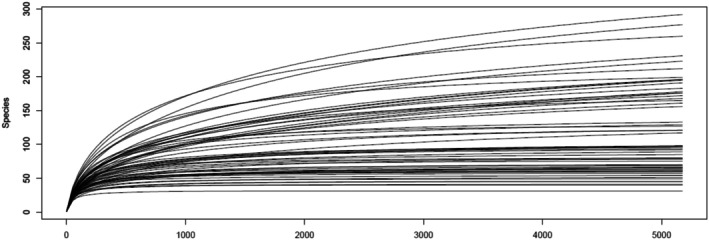
Rarefaction Curves for Archaeal and Bacterial Communities Based on ASV richness per sample. Each curve represents a sample standardized to the same sequencing depth. The *x*‐axis indicates the number of sequences subsampled, and the *y*‐axis shows the corresponding number of observed ASVs.

First, all ASVs unassigned at the phylum level and non‐prokaryotic ASVs (i.e., ASVs identified as “Eukaryota”, “Chloroplast” and “Mitochondria” at phylum, order and family levels, respectively) were removed from the data set. From this ASV‐level matrix, all ASVs were annotated and merged considering their finest taxonomic classification, using the fantastic R package (Teunisse 2022). Before further analyses, ASV counts were normalized by rarefying considering the sample with the smallest number of sequences (5608 sequences) (Appendix 3: Figure A1). Finally, the global data set was divided into the two subcommunities, ‘ANME‐SRB’ and ‘Other’. For this, ASVs belonging to the phylum Desulfobacterota and the classes ANME‐1 and Methanosarcina (taxonomic classifications according to SILVA database) were separated from the total data set, which together made up the ANME‐SRB matrix.

Alpha diversity analyses were performed by measuring Chao1 richness index and Inverse Simpson (InvSimpson) diversity index with the phyloseq R package (McMurdie and Holmes 2013). To evaluate significant differences in alpha diversity measurements in relation to the different areas (A, C and E) and sediment layers (within or below SMTZ), Kruskal–Wallis and Wilcoxon signed rank tests were performed and plotted using the functions *compare_means* and *ggviolin*, respectively, through the ggpubr R package (Kassambara 2018). To explore significant associations between alpha diversity and geochemical gradients, Spearman correlations were calculated and plotted using the corrplot R package (Wei et al. 2017), which were deemed statistically significant if *p* < 0.05.

Beta diversity analyses were carried out based on Bray–Curtis dissimilarity matrices using the R package vegan (Oksanen et al. 2013) and phyloseq (McMurdie and Holmes 2013), and plots were drawn using the ggplot2 R package (Villanueva and Chen 2019). Composition difference and heterogeneity between areas (A, C, and E) and sediment layers (within and below SMTZ) were tested with Permutation Multivariate Analysis of Variance (PERMANOVA; Anderson 2001) and Permutational Analysis of Multivariate Dispersion (PERMDISP; Anderson 2006), respectively, and visualized with Nonmetric Multidimensional Scaling (NMDS). PERMANOVA, PERMDISP, and NMDS were run using the functions *adonis2*, *betadisper*, and *ordinate*, respectively. Additionally, a Similarity Percentage (SIMPER) analysis was applied to identify the taxa that most contributed to compositional dissimilarities between sampling areas and sediment layers. SIMPER was performed separately for the ANME‐SRB consortium and for the remaining prokaryotic community using Bray‐Curtis dissimilarities based on relative abundance data. The influence of the geochemical gradients on composition difference was measured with distance‐based Redundancy Analysis (dbRDA; Oksanen et al. 2007) using the *capsale* function. First, the environmental data matrix was *z*‐score transformed, and variance inflation factors of 0.10, representing covariability, were discarded before stepwise model selection using the *ordistep* function (both directions). Finally, a final dbRDA was performed and significant variables were added to the ordination as arrows. Analysis of variance (ANOVA; *p* < 0.05) was used to test the significance of dbRDA models.

The portion of unique and shared ASVs between areas (A, C, and E) and sediment layers (within and below SMTZ) was verified and visualized using the MiscMetabar R package (Taudière 2023) on absolute abundance data. The core microbiome was determined using the microbiome R package (Lahti and Shetty 2018) on compositional abundance data considering a prevalence of 80% at 2% relative abundance. Functional annotations of the cold seeps microbiota were predicted using the tool Functional Annotation of Prokaryotic Taxa (FAPROTAX v. 1.2.10), a database that converts microbial community profiles into putative functional profiles based on cultivated strains (Louca et al. 2016). The FAPROTAX results were plotted as a heatmap, after discarding the functions identified in fewer than 10 reads and normalizing the read counts to the total function identifications for each sample, using the pheatmap R package (Kolde and Kolde 2015). The relative abundance of the 10 most abundant phyla and genera was accessed using phyloseq (McMurdie and Holmes 2013) and plotted using the microViz R package (Barnett et al. 2021) with the *comp_barplot* function. All the community analyses were run in R (v. 4.3.3) (R Core Team 2021).

## 3 Results

### 3.1 Archaeal and Bacterial Richness and Diversity

After the quality filtering, a total of 6,277,541 reads (details in Appendix 2: Table A2) were used for downstream analyses. After removing all ASVs not classified at the phylum level and classified as Eukarya, Chloroplast, and Mitochondria, a total of 16,662 ASVs were retained, which were merged (considering the best hit taxonomic classification) into 967 prokaryotic taxa.

**FIGURE 2:**
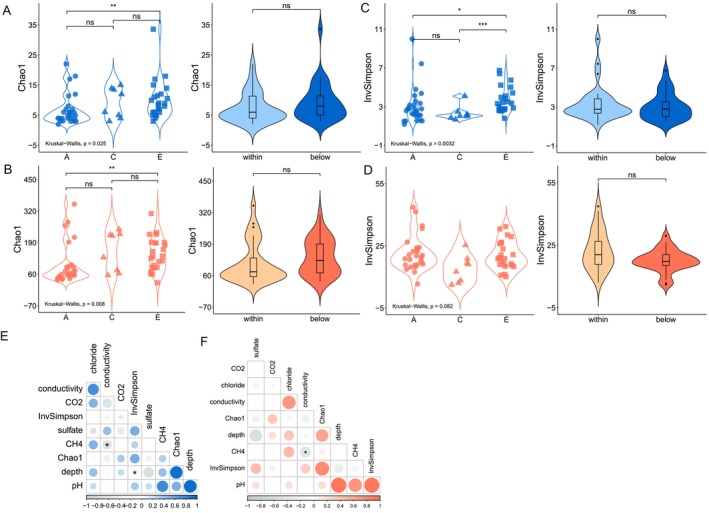
Alpha diversity of archaeal and bacterial communities and potential geochemical drivers. Chao1 and Inverse Simpson indexes of sample groups based on sampling areas (A, C and E) and sediment layer (within and below SMTZ) for ANME‐SRB (A and C) and Other (B and D). Statistical differences between groups are indicated by asterisks (*p < 0.01; **p < 0.05; ***p < 0.001). Spearman correlations between alpha diversity indexes and geochemical variables for ANME‐SRB (E) and Other (F). Statistical significance was computed for all pairwise comparisons (*p* < 0.05) and are shown as black asterisks.

When comparing the different areas (A, C, and E), significant differences in richness (Chao1 index) were observed for both groups (ANME‐SRB, *p* = 0.025; Other, *p* = 0.008) (Figure 2A,B). Considering the diversity (InvSimpson index), significant differences for ANME‐SRB were observed (*p* = 0.003), while no differences were detected for Other (*p* = 0.082) (Figure 2C,D).

**FIGURE A2:**
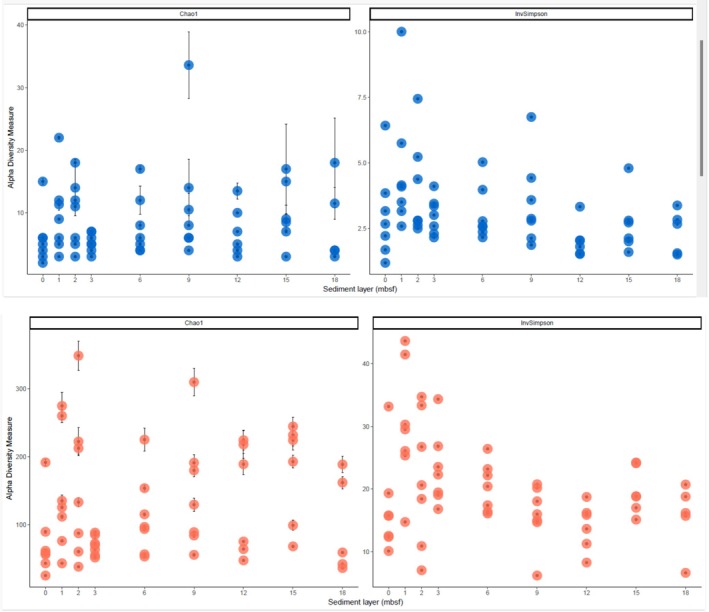
Chao1 and inverse Simpson diversity indices for sample groups categorized by sediment depth (ranging from 1 to 18 meters below the seafloor) for ANME‐SRB (blue) and other (orange).

No differences in alpha diversity were observed when comparing the sediment layers (within and below SMTZ) for both ANME‐SRB and Other (Figure 2). The correlations between alpha diversity metrics and geochemical variables did not show any significant relationship for both groups (Figure 2E,F), although there was a trend towards decreasing diversity (InvSimpson index) with increasing depth for both ANME‐SRB and Other (Appendix 4: Figure A2).

### 3.2 Community Composition and Spatial Heterogeneity

**FIGURE 3:**
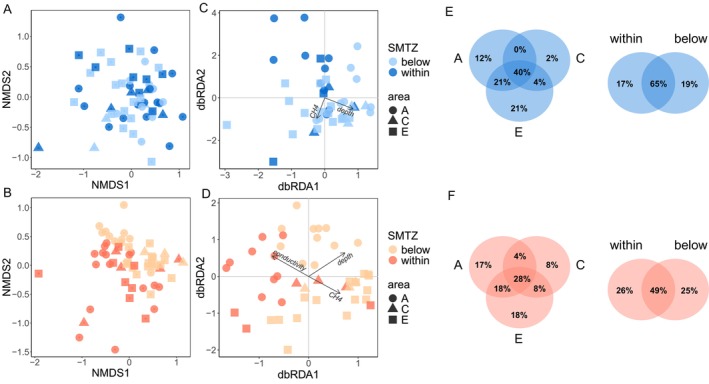
Beta diversity of microbial communities and the influence of geochemical gradients. Nonmetric multidimensional scaling (NMDS) using Bray–Curtis dissimilarity values for ANME‐SRB (A) and Other (B) and distance‐based redundancy analysis (dbRDA) showing the relationships of archaeal and bacterial communities with geochemical variables for ANME‐SRB (C) and Other (D). Dot colors represent the sediment layer (within and below SMTZ) and different shapes represent the sampling areas (A, C, and E). Venn diagrams showing unique and shared archaeal and bacterial taxa among areas and sediment layers for ANME‐SRB (E) and Other (F).

Dissimilarities in archaeal and bacterial communities using NMDS plots based on Bray‐Curtis distances showed distinctions in prokaryotic composition between sediment layers (within and below SMTZ) and areas (A, C, and E) for both ANME‐SRB and Other (Figure 3A,B). These differences were confirmed by PERMANOVA, in which both factors (area and SMTZ) significantly influenced the community composition for both components (Table 1). SIMPER analyses revealed that differences in the ANME‐SRB community were primarily driven by variations in sulfate‐reducing bacteria and methanogenic archaeal lineages across areas and sediment layers. For Other, compositional differences were mainly associated with deeply branching and uncultured taxa, including JS1, *Sulfophobococcus* and Lokiarchaeia (Appendix 5: Table A3). dbRDA based on Bray‐Curtis distances showed that for ANME‐SRB, community composition was significantly associated with depth and CH_4_ (*p* = 0.005) (Figure 3C). For Other, community composition was significantly associated with depth (*p* = 0.001), CH_4_ (*p* = 0.002), and conductivity (*p* = 0.019) (Figure 3D).

**TABLE 1:** PERMANOVA to detect differences and PERMDISP to detect dispersion (variability) in ASV composition, considering the ANME‐SRB and Other groups, among areas (A, C, and E) and sediment layers (within and below SMTZ). Significant tests (*p* < 0.05) are in bold.

Community	Factor	PERMANOVA			PERMDISP	
		*R* ^2^	Pseudo‐*F*	*p*	*F*	*p*
ANME‐SRB	Area	0.084	2.822	**< 0.001**	0.500	0.588
	SMTZ	0.056	3.395	**< 0.001**	1.436	0.221
	Area*SMTZ	0.030	0.922	0.470		
	Residual	0.828				
Other	Area	0.112	3.843	**< 0.001**	0.705	0.512
	SMTZ	0.066	4.484	**< 0.001**	6.045	0.120
	Area*SMTZ	0.035	1.163	0.192		
	Residual	0.785				

Of the ASVs belonging to ANME‐SRB, 35% were exclusive to a particular area (A, C, or E) while 40% were shared among the three locations. Regarding the sediment layer, 35% ASVs have occurred only within or below SMTZ, while 65% were shared between the two habitats (Figure 3E). Considering the ASVs belonging to Other, 43% were exclusive to a particular area (A, C, or E) while 28% were shared among the three locations. In relation to the sediment layer, 51% ASVs have occurred only within or below SMTZ, while 49% were shared between the two habitats (Figure 3F). The core microbiome analysis showed that 12 ASVs were widespread throughout the samples (considering 80% prevalence at 2% relative abundance), which together represented 41.2% of the archaeal and bacterial abundance considering the total data set (Table 2).

**TABLE 2:** ASVs retained by the core microbiome analysis considering 80% prevalence at 2% relative abundance, and their relative abundances in the total data set.

	Taxonomy			Relative abundance (%)
	Domain	Phylum	Taxa^a^	
*ASV5*	Archaea	Crenarchaeota	*Sulfophobococcus*	9.5
*ASV3*	Bacteria	Caldatribacteriota	JS1 (Class)	5.9
*ASV1*	Bacteria	Aerophobota	Aerophobales (Order)	5.1
*ASV17*	Archaea	Asgardarchaeota	Lokiarchaeia (Class)	4.0
*ASV119*	Archaea	Nanoarchaeota	SCGCAAA011‐D5 (Family)	3.3
*ASV136*	Bacteria	Proteobacteria	*Candidatus* Thiophysa	2.8
*ASV186*	Archaea	Halobacterota	*Candidatus* Haloectosymbiotes	2.7
*ASV33*	Archaea	Crenarchaeota	*Candidatus* Nitrosopumilus	2.7
*ASV144*	Archaea	Crenarchaeota	Bathyarchaeia (Class)	1.7
*ASV336*	Bacteria	Planctomycetota	SG8‐4 (Family)	1.4
*ASV22*	Bacteria	Proteobacteria	*Methylobacterium‐Methylorubrum*	1.1
*ASV248*	Bacteria	Desulfobacterota	*Desulfatiglans*	1.0

^a^ Considering the finest taxonomic resolution.

**FIGURE 4:**
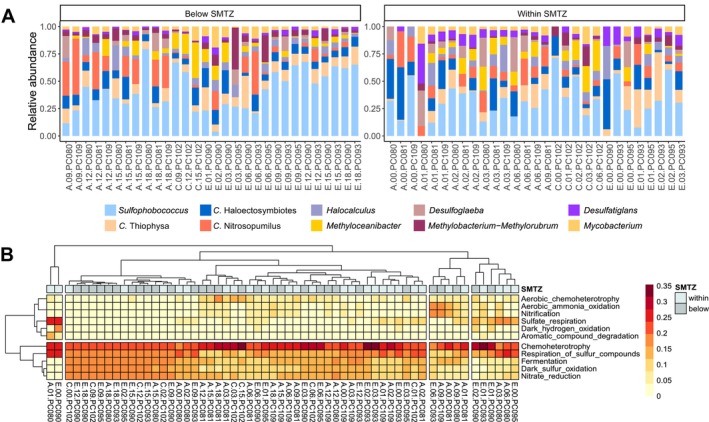
Taxonomic and functional community composition. Relative abundance of major archaeal and bacterial genera (A) in each deep‐sea sediment sample analyzed in the northeastern flank of the Rio Grande Cone (Pelotas Basin), southern Brazil. The samples are ordered by sampling areas (A, C, and E) and sediment layer (within and below SMTZ). Heatmap of metabolic functions of archaeal and bacterial taxa predicted through FAPROTAX (B). The data is based on ASVs occurrence (number of ASVs capable for each function).

At the phylum level, 80 phyla or candidate divisions were identified, of which 18 were found in abundance higher than 1% of the total sequences in the data set. The 5 most abundant phyla were Crenarchaeota (15.1%), Proteobacteria (11.2%), Chloroflexi (11.1%), Halobacterota (9.1%), and Caldatribacteriota (6.7%), and the 10 most abundant phyla corresponded to 76.6% of the total archaeal and bacterial abundance (Figure 4A). At the genus level, 706 genera or candidates were recorded, of which 11 were found in abundance higher than 1% of the total sequences in the data set. The 5 most abundant genera were *Sulfophobococcus* (9.5%), *Candidatus* Thiophysa (2.8%), *Candidatus* Haloectosymbiotes (2.7%), *Candidatus* Nitrosopumilus (2.7%), and *Halocalculus* (2%), and the 10 most abundant identified genera corresponded to 24.2% of the total archaeal and bacterial abundance (Figure 4A).

The group ANME‐SRB was composed of 60 ASVs belonging to 30 identified genera (25 from SRB and 5 from ANME), the most abundant of which were *Desulfoglaeba* (1.2%), *Desulfatiglans* (1%), and the ANME‐1b group (0.5%). Archaeal and bacterial taxa were functionally annotated using the FAPROTAX database, which resulted in 91 assigned ecological functions. These functions included chemoheterotrophy (accounting for 14.6% abundance), respiration of sulfur compounds (10.5%), fermentation (9.4%), and nitrate reduction (8.9%). Chemoheterotrophy and respiration of sulfur compounds were the dominant metabolic archaeal and bacterial functions, and there was no clear grouping of samples according to area (A, C, and E) and sediment layer (within and below SMTZ) (Figure 4B).

## 4 Discussion

The Rio Grande Cone deep‐sea sediment is a newly described environment that presents geological and geochemical features that can sustain abundant chemosynthetic communities (Miller et al. 2015; Rodrigues et al. 2017; Giongo et al. 2016; Medina‐Silva et al. 2018). Here, we tested three main hypotheses: (i) that sediment layers related to the sulfate–methane transition zone (SMTZ) would be the primary drivers of prokaryotic community composition; (ii) that methane and sulfate gradients would exert a stronger influence on the ANME–SRB subcommunity than on the remaining prokaryotic assemblage; and (iii) that depth‐related geochemical gradients would be reflected in stratified metabolic profiles. Overall, our results partially corroborated the first two hypotheses, as both sediment layer and geographic area significantly structured community composition and methane emerged as a key driver for both ANME‐SRB and other taxa, whereas the third hypothesis was not supported, since predicted metabolic profiles showed no clear stratification with depth or SMTZ position. These findings provide the framework for the discussion below, in which patterns of diversity, composition, and functional potential are examined in detail.

### 4.1 Trends in Prokaryotic Richness and Diversity

The alpha diversity results showed differences in richness (Chao1 index) and diversity (Inverse Simpson index) when comparing areas, while no significant differences were observed when comparing the sediment layers, for both ANME‐SRB and Other (Figure 2). The differences in richness occurred between areas A and E, in which the latter presented higher ASV richness compared to the former (Figure 2). Miller et al. (2015) presented detailed geological and geochemical characteristics of areas A and E and showed that extensive pockmark fields were found in both areas, in which piston core samples exhibited several gas hydrate layers interbedded with muddy sediments and putative chemosynthetic organisms. These data point to the widely recognized establishment of chemosynthesis‐based seep communities on the seafloor sustained by the upward flux of methane from hydrates (Pohlman et al. 2009). Nonetheless, so far, there are no clear environmental differences that could explain the difference in bacterial and archaeal richness between the surveyed areas in this study.

Although there were no significant differences in alpha diversity with respect to the sediment layers related to the SMTZ (Figure 2), a trend was observed in which richness and diversity decreased with increasing sedimentary depth (Appendix 4: Figure A2). Previous studies have also shown a declining trend in cell abundance and/or species richness from shallower layers to deeper layers of marine sediments (Inagaki et al. 2006; Harrison et al. 2009; Mills et al. 2012; Ciobanu et al. 2014; Walsh et al. 2016; Hoshino et al. 2020). The decrease in bacterial and archaeal richness with increasing sediment depth was also observed recently in a study with a large dataset spanning sediment depths of 0.1–678 m (Hoshino et al. 2020), indicating that this is a general pattern in marine sediments. This pattern is largely explained by the selection of taxa adapted to the anoxic and nutrient‐poor conditions of the deep layers of marine sediment when compared to sediments located at the sediment–water interface (Hoehler and Jørgensen 2013). In our study, however, there were no significant correlations between geochemical gradients and richness and diversity indices, either for ANME‐SRB or Other (Figure 2), which can be explained by the action of other unmeasured environmental variables, such as oxygen availability, and ecological factors, such as biotic interactions, on prokaryotic diversity.

### 4.2 Archaeal and Bacterial Composition Across Areas and Sediment Layers

The beta diversity results showed that the area and the sediment layer are important factors shaping archaeal and bacterial composition (Figure 3; Table 1; Appendix 5: Table A3). The composition variation of prokaryotic taxa in different marine sediment layers is largely reported (Mills et al. 2012; Guardiola et al. 2015; Inagaki et al. 2015; Walsh et al. 2016), including distinct layers related to the SMTZ in cold seep ecosystems (Harrison et al. 2009; Zhong et al. 2023; Inagaki et al. 2015). The surface layer of marine sediment is exposed to mineral and biotic particle accumulation (sinking) from the water column, which results in particular physicochemical characteristics and biotic composition. This environmental condition explains, for example, the typical presence of some photosynthetic organisms in marine sediments, so far from the photosynthetic zone (Treude et al. 2014; Carr et al. 2015; Guardiola et al. 2015). Previous studies have also shown that part of the microbial community of marine sediments is made up of a subgroup of taxa derived from the water column, highlighting the importance of dispersal throughout the pelagic zone (Walsh et al. 2016).

In relation to the SMTZ in cold seeps, specifically, it is known that the availability of both methane and sulfate forms a specific niche for certain prokaryotic taxa, resulting in a distinct microbial composition when compared to other sediment layers (Boetius et al. 2000; Harrison et al. 2009). The distribution of the ANME and SRB groups in the SMTZ is widely studied (Yanagawa et al. 2011; Biddle et al. 2012; Vigneron et al. 2013; Beulig et al. 2019), which consume methane and sulfate simultaneously in the SMTZ due to the anaerobic oxidation of methane (AOM; Boetius et al. 2000; Orphan, House, et al. 2001, Orphan, Hinrichs, et al. 2001), a process with important implications in deep‐sea sulfur cycling (Li et al. 2021) and control of methane emissions (Reeburgh 2007). In this context, we hypothesized that the distinct sediment layers in relation to the SMTZ would be the main driver of archaeal and bacterial composition. This hypothesis was partially corroborated, since the distinct surveyed areas (A, C, and E) also showed significant importance (and greater, considering the *R* ^2^ values) in shaping the community composition (Table 1). The distinct prokaryotic composition between the areas may be due to different environmental conditions not directly evaluated in this study, such as the depth of the water column, geological features, and the presence/abundance of other biological groups, such as microeukaryotes and metazoans.

The fact that the non‐ANME‐SRB community was also structured by the sediment layer (Table 1) suggests the influence of additional geochemical gradients and environmental factors beyond the distinction delimited by the SMTZ, which is corroborated by the significant influence of the conductivity observed in the dbRDA analysis (Figure 3). In fact, previous studies have shown that bacterial and archaeal taxa other than ANME and SRB are differently distributed in sediment layers related to the SMTZ. Harrison et al. (2009), for instance, observed that the abundance of some taxa, such as Planctomycetes, candidate division JS1 (Atribacteria), and Actinobacteria, varied across the SMTZ horizon. A recent detailed characterization in deep‐sea sediment layers related to the SMTZ also showed such differences (Metze et al. 2023), in which a Chloroflexota‐dominated community above the upper SMTZ was changed by a Proteobacteria‐dominated community below the secondary SMTZ. In our study, spatial variations at the phylum level can also be observed, such as a higher abundance of Chloroflexi and Planctomycetota in the most superficial sediments (within the SMTZ), while Caldatribacteriota and Aerophobota presented the opposite pattern (Figure 4A). Along with the fact that most ASVs were exclusive to each sediment layer (Figure 3), these findings point to a taxonomically diverse community formed by generalist and specialist taxa with different environmental preferences.

Our second hypothesis predicted that, among the geochemical variables, methane and sulfate would be more important for ANME‐SRB when compared to the remaining prokaryotic community. The dbRDA results have shown a significant impact of the methane and depth for both groups, and conductivity for Other (Figure 3), while sulfate probably did not make it into the final model due to its correlation with methane. These geochemical gradients have already been identified as important ecological drivers of prokaryotic taxa in cold seeps (Roalkvam et al. 2012; Green‐Saxena et al. 2014; Ruff et al. 2015; Cruaud et al. 2017; Semler et al. 2022; Dong et al. 2023), highlighting the environmental filtering as an important ecological process in shaping cold seeps prokaryotic communities. Furthermore, the indication of methane as a significant predictor of archaeal and bacterial composition for both ANME‐SRB and Other confirms the crucial role of this compound in the formation and maintenance of cold seeps dynamics. Finally, the significant influence of depth in shaping prokaryotic composition for both ANME‐SRB and Other indicates the role of other depth‐structured geochemical gradients not measured in our study. For example, the influence of hydrogen sulfide, which derives from the sulfate metabolism, affects the distribution of clade ANME‐2 so that their abundance decreases with increasing hydrogen sulfide concentration (Meulepas et al. 2009; Roalkvam et al. 2012).

### 4.3 The “cold seep microbiome”

The core microbiome analysis showed that 12 ASVs were widespread throughout the samples (considering 80% prevalence at 2% relative abundance), which together represented 41.2% of the archaeal and bacterial abundance considering the total data set (Table 2). The core microbiome was formed by taxa typically associated with the deep ocean environment and cold seeps, including the class Bathyarchaeia and the genera *Sulfophobococcus* and *Candidatus* Nitrosopumilus from the Crenarchaeota archaeal phylum, and the bacterial lineage JS1, which are included in the previously documented “seep microbiome” (Inagaki et al. 2006; Carr et al. 2015; Oni et al. 2015; Ruff et al. 2015). The third and fourth most abundant ASVs retained in the core microbiome are members of the bacterial order and archaeal class Aerophobales and Lokiarchaeia, respectively (Table 2), which are commonly found in deep ocean sediments and cold seeps (Jiang et al. 2022; Metze et al. 2023). Lokiarchaeota is a proposed archaeal phylum (Lambert 2019) and includes all members of the group previously named Deep Sea Archaeal Group, also known as Marine Benthic Group B (Caceres et al. 2019).

All ASVs identified at the genus level (or candidate) that comprised the core microbiome are among the 10 most abundant genera in the total dataset (Figure 4), and trends with sediment depth could be observed for some of these taxa. In this context, we can highlight an increase in abundance with the depth of *Sulfophobococcus* and *Candidatus* Nitrosopumilus, the latter being more prevalent in the deeper sediment layers of area A (Figure 4). The sulfate‐reducing genera *Desulfoglaeba* and *Desulfatiglans* from the bacterial phylum Desulfobacterota, on the other hand, were more associated with shallow sediments within the SMTZ (Figure 4). These bacterial genera were proposed to be in the newly proposed phylum Thermodesulfobacteriota (Waite et al. 2020), characterized by a sulfate‐associated metabolism and prevalence in deep‐sea habitats.

It is worth mentioning that the most abundant taxon in our study system was the archaeal genus *Sulfophobococcus*, which was dominant in all sampled areas and sediment layers (Figure 4). *Sulfophobococcus* belongs to the class Thermoprotei and is characterized as an anaerobic (hyper)thermophilic archaeum (Hensel et al. 1997), typically found in deep‐sea sediments and hydrothermal vents, and terrestrial geothermal springs (Mueller et al. 2021; Li et al. 2022; Lai et al. 2023), rather than in the “seep microbiome” (Ruff et al. 2015).

The atypical dominance of *Sulfophobococcus* across all sampled sites highlights a key knowledge gap regarding its ecological role in cold seep systems. In this context, future genomic sequencing would be essential to resolve the ecogenomics of *Sulfophobococcus* populations in this system, enabling the identification of genes involved in its metabolism and allowing direct comparison with thermophilic relatives from hydrothermal and geothermal environments. Such analyses would clarify whether the dominance of *Sulfophobococcus* reflects active metabolic specialization within the seep environment or a broader ecological versatility that remains cryptic in amplicon‐based surveys.

### 4.4 Stratification of Metabolic Profiles

Our third hypothesis predicted that distinct metabolic profiles would be observed throughout the sediment layers, as a result of depth‐structured geochemical gradients influencing the prokaryotic metabolism. In our FAPROTAX results, however, no clear‐cut differences according to sediment depth were detected (Figure 4). In the subseafloor, general patterns of depth‐structured metabolic profiles are known. Among these, it has long been recognized that sulfate reduction tends to be the dominant form of microbial respiration when sulfate is not completely depleted, whereas methanogenesis is the dominant form of microbial respiration in deeper sediment layers when sulfate is depleted (Reeburgh 1980). This pattern was recently demonstrated by Metze et al. (2023), showing that the chemical stratification coincided with the stratification of prokaryotic composition shifts and biological functions in deep‐sea sediment layers related to the SMTZ.

The FAPROTAX results showed the chemoheterotrophy and respiration of sulfur compounds as the dominant predicted functions (based on the abundance of taxa performing such functions) and that, in general, functions involved in sulfur and nitrogen cycles were dominant (Figure 4). The heatmap, however, did not show a structure of metabolic profiles related to the sediment layers or the sampled geographic areas (Figure 4), indicating that the most prevalent predicted metabolic functions are widely distributed in our study system. Moreover, although methane‐related functions were recorded (e.g., methanotrophy and methanogenesis), these were less expressive considering the total data set (data not shown). These results are consistent with studies showing that chemoheterotrophy and functions associated with sulfur and nitrogen were the main potential metabolic processes of bacterial and archaeal communities in marine cavern waters (He et al. 2019) and deep‐sea sediments (Fu et al. 2020), as well as sediments and seawater from a cold seep (Zhong et al. 2023).

Notably, the presence of taxa associated with nitrate reduction (Figure 4) raises the possibility that alternative methane oxidation pathways may operate in this system. In addition to the AOM mediated by ANME–SRB consortia, nitrate‐dependent anaerobic oxidation of methane (n‐DAMO) has been described in diverse marine and freshwater environments (Raghoebarsing et al. 2006; Haroon et al. 2013). Although n‐DAMO was not explicitly targeted in this study, the detection of nitrate‐reducing functional groups, combined with the complex redox gradients expected in deep‐sea seep sediments, suggests that the geochemical conditions of the Rio Grande Cone could potentially support n‐DAMO‐related processes.

Finally, two main methodological limitations of this study should be acknowledged. First, community composition was inferred from a single hypervariable region of the 16S rRNA gene (V3‐V4), which, although widely used, may not provide equivalent taxonomic resolution for archaeal and bacterial lineages and may bias relative diversity estimates in complex deep‐sea assemblages. Second, functional profiles were inferred using FAPROTAX, which assigns metabolic functions based on phenotypic information from predominantly cultured taxa and therefore represents putative rather than directly measured functional potential, a limitation that is particularly relevant in deep‐sea sediments dominated by uncultured microorganisms. Consequently, the absence of clear depth‐structured metabolic profiles should be interpreted with caution. Future studies integrating shotgun metagenomics or full‐length 16S rRNA sequencing would allow direct reconstruction of metabolic pathways and genomes, providing higher‐resolution insights into functional stratification and ecological roles across the SMTZ and adjacent sediment layers.

## 5 Conclusions

Our results showed that, at the studied cold seep site, both ANME‐SRB and other communities presented a heterogeneous taxonomic composition across the different surveyed geographic areas and sediment layers related to the SMTZ. Moreover, differences in methane concentrations along sediment depth were the main predictors of bacterial and archaeal composition, evidencing the importance of local environmental conditions on prokaryotic distribution. Functional predictions revealed that bacterial and archaeal communities are largely involved in the sulfur and nitrogen element cycles. Unlike taxonomic composition, however, no distinct metabolic profiles structured by area or sediment layer were observed. The main groups associated with deep‐ocean habitats and cold seeps were recorded, evidencing the existence of a typical “seep microbiome”. These results shed light on yet undescribed roles played by microbial communities in the dynamics of biogeochemical cycles occurring in a methane cold seep area at Western South Atlantic recently discovered.

## Author Contributions

**Renata Medina‐Silva:** conceptualization (equal), data curation (equal), investigation (lead), project administration (equal), resources (equal), validation (equal), writing – review and editing (equal). **Karine A. Felix Ribeiro:** data curation (equal), formal analysis (equal), methodology (equal), validation (equal), writing – original draft (equal), writing – review and editing (equal). **Taiz L. Lopes Simão:** conceptualization (equal), data curation (equal), formal analysis (equal), methodology (equal), writing – original draft (equal). **Eduardo Eizirik:** conceptualization (equal), data curation (equal), funding acquisition (lead), methodology (equal), project administration (equal), resources (equal), supervision (equal), validation (equal). **Raquel Dias:** formal analysis (equal), investigation (equal), methodology (equal), validation (equal), visualization (equal). **Eric W. Triplett:** formal analysis (equal), funding acquisition (equal), investigation (equal), methodology (equal), resources (equal), supervision (equal), validation (equal). **Adriana Giongo:** conceptualization (equal), data curation (equal), formal analysis (equal), investigation (equal), methodology (equal), supervision (equal), validation (equal), writing – review and editing (equal). **João M. M. Ketzer:** conceptualization (equal), funding acquisition (equal), investigation (equal), methodology (equal), project administration (equal), resources (equal), visualization (equal). **Adolpho H. Augustin:** data curation (equal), formal analysis (equal), investigation (equal), methodology (equal), visualization (equal), writing – original draft (equal). **Luiz F. Rodrigues:** data curation (equal), formal analysis (equal), investigation (equal), methodology (equal), validation (equal), visualization (equal). **Dennis J. Miller:** conceptualization (equal), funding acquisition (equal), methodology (equal), project administration (equal), resources (equal), validation (equal). **Adriano R. Viana:** conceptualization (equal), funding acquisition (equal), methodology (equal), project administration (equal), resources (equal), validation (equal).

## Funding

This work was supported by Conselho Nacional de Desenvolvimento Científico e Tecnológico, Petrobras.

## Conflicts of Interest

The authors declare no conflicts of interest.

## Data Availability

The sequencing data generated in this study are available in the NCBI Sequence Read Archive (SRA) under the BioProject accession number PRJNA1266721 and can be accessed at https://www.ncbi.nlm.nih.gov/sra/PRJNA1266721. The associated metadata, including geochemical and sampling information, is available in the main text and Appendices 1, 2, 3, 4, 5.

## Appendix 1

**TABLE A1:** Geographic and geochemical data of the deep‐sea sediment samples from the northeastern flank of the Rio Grande Cone (Pelotas Basin), Southern Brazil.

Sample ID	Area	SMTZ	Depth (mbsf^a^)	Chloride	Sulfate	CO_2_	CH_4_	pH	Conductivity
C.00.PC102	C	Within	0	20,043.8	0	*NA*	*NA*	8.09	43.35
C.01.PC102	C	Within	1	11,891.2	1382	*NA*	*NA*	8.01	43.07
C.02.PC102	C	Within	2	20,644.4	0	277.75	18.4	7.88	42.68
C.03.PC102	C	Within	3	18,713	1543.6	705.06	23.3	7.84	42.73
C.06.PC102	C	Within	6	19,783.8	739.6	1680.24	27.91	7.79	43.8
C.09.PC102	C	Below	9	11,027.2	0	2201.64	14,656.99	7.01	44.31
C.12.PC102	C	Below	12	19,886.4	0	8253.23	86,522.01	7.53	43.81
C.15.PC102	C	Below	15	21,094.2	0	13,571.98	77,984.74	7.47	43.74
A.00.PC109	A	Within	0	20,310	2782.2	*NA*	*NA*	7.95	47.13
A.01.PC109	A	Within	1	21,407.8	2911.6	*NA*	*NA*	7.79	46.41
A.02.PC109	A	Within	2	18,640.2	3555.4	355.85	7866.2	7.93	46.46
A.03.PC109	A	Within	3	18,828	2242	323.48	12,776.28	7.83	45.25
A.06.PC109	A	Within	6	20,508.6	2385	350.89	4392.66	7.95	45.31
A.09.PC109	A	Below	9	19,658.8	0	133.41	20,630.24	8.14	43.46
A.12.PC109	A	Below	12	20,641.4	0	179.59	30,835.68	8.2	44.19
A.15.PC109	A	Below	15	21,393.8	0	1852.76	60,132.03	7.86	45.85
A.18.PC109	A	Below	18	20,710.4	0	685.06	21,918.41	7.87	46.73
A.00.PC080	A	Within	0	20,188.6	2344	*NA*	*NA*	8	48.51
A.01.PC080	A	Within	1	19,144.6	1745.6	*NA*	*NA*	7.9	44.51
A.02.PC080	A	Within	2	19,901.4	1467.2	333.55	2745.19	7.78	48.45
A.03.PC080	A	Within	3	20,469.2	1032.8	212.29	2376.08	7.95	47.27
A.06.PC080	A	Within	6	19,494.2	493.2	225.32	2559.56	7.94	49.3
A.09.PC080	A	Below	9	22,178.2	0	166.95	14,920.47	7.99	41.18
A.12.PC080	A	Below	12	20,115.6	0	387.48	21,863.89	8.21	47.05
A.15.PC080	A	Below	15	19,695.2	0	502.31	27,646.78	8.13	49.95
A.18.PC080	A	Below	18	21,600.8	107.4	544.89	24,061.84	7.91	40.28
A.00.PC081	A	Within	0	21,413.4	2532.6	*NA*	*NA*	8.27	48.42
A.01.PC081	A	Within	1	22,223.6	2557.8	*NA*	*NA*	8.12	45.04
A.02.PC081	A	Within	2	21,389	2449.2	231.07	1908.05	8.17	47.49
A.03.PC081	A	Within	3	21,745.4	2813.8	165.51	1881.25	8.07	46.39
A.06.PC081	A	Within	6	19,273.4	1504	566.88	1922.37	8	47.74
A.09.PC081	A	Within	9	20,265.8	321.4	791.24	2092.24	7.94	47.59
A.12.PC081	A	Below	12	18,907.4	0	364.82	40,427.44	8.16	45.6
A.15.PC081	A	Below	15	20,945.4	0	622.67	46,649.1	8.11	44.65
A.18.PC081	A	Below	18	21,838.8	0	1451.29	29,825.78	7.9	45.52
E.00.PC090	E	Within	0	20,119.8	107.6	*NA*	*NA*	8.23	42.5
E.01.PC090	E	Below	1	20,132.6	0	*NA*	*NA*	8.28	41.59
E.02.PC090	E	Below	2	19,825.6	0	2751.22	74,504.14	8.41	41.57
E.03.PC090	E	Below	3	20,414	0	1472.82	57,064.5	8.44	39.68
E.06.PC090	E	Below	6	20,746	95	782.43	73,562.65	8.13	42.56
E.09.PC090	E	Below	9	20,926.4	0	1151.66	47,525.9	8.41	42.27
E.12.PC090	E	Below	12	19,793.4	0	448.84	19,809.57	8.47	39.94
E.15.PC090	E	Below	15	21,178	0	2515.45	26,935.35	8.28	41.53
E.18.PC090	E	Below	18	21,926.2	0	1857.58	25,487.58	8.17	41.43
E.00.PC093	E	Within	0	19,931	2396	*NA*	*NA*	7.92	42.67
E.01.PC093	E	Within	1	18,725.6	2159.6	*NA*	*NA*	7.66	42.6
E.02.PC093	E	Within	2	19,346	2169.6	724.73	3706.15	7.8	43.22
E.03.PC093	E	Within	3	19,100	1682.6	3993.41	2931.09	7.7	43.24
E.06.PC093	E	Below	6	19,170.8	0	3237.49	54,252.88	7.85	38.8
E.09.PC093	E	Below	9	19,553.8	0	3103.85	127,831.56	7.89	38.11
E.12.PC093	E	Below	12	20,260	0	3845.95	128,468.89	7.93	39.31
E.15.PC093	E	Below	15	20,612.4	0	4885.84	121,164.53	7.72	39.62
E.18.PC093	E	Below	18	21,184.2	0	3957.17	68,213.83	7.79	39.39
E.00.PC095	E	Within	0	16,472.4	62.8	*NA*	*NA*	8	38.16
E.01.PC095	E	Within	1	18,243.6	191.8	*NA*	*NA*	7.92	39.45
E.02.PC095	E	Within	2	14,946.8	554.8	4021.87	92,132.71	7.99	34.32
E.03.PC095	E	Below	3	22,164	84	2709.9	58,568.12	7.94	49.66
E.06.PC095	E	Below	6	18,810.4	0	3470.89	67,104	7.98	40.86
E.09.PC095	E	Below	9	18,947.6	0	2841.12	28,678.52	7.96	41.76

^a^ Meters below seafloor.

## Appendix 2

**TABLE A2:** Sequencing data throughout the steps of filtering, denoising, and chimera removal.

Sample ID	Input	Filtered	Denoisedf	Nonchim
C.00.PC102	27,520	12,520	9020	9020
C.01.PC102	31,544	17,516	113,36	11,142
C.02.PC102	199,044	160,531	153,069	129,406
C.03.PC102	41,099	22,445	17,422	17,053
C.06.PC102	84,589	69,517	65,885	58,963
C.09.PC102	94,395	78,910	72,280	61,704
C.12.PC102	244,054	202,577	184,846	160,623
C.15.PC102	234,260	195,465	182,486	167,032
A.00.PC109	137,443	98,886	70,610	70,610
A.01.PC109	479,430	217,665	197,941	183,945
A.02.PC109	283,986	137,727	120,891	117,280
A.03.PC109	158,249	114,952	95,161	93,387
A.06.PC109	85,228	61,077	54,474	53,179
A.09.PC109	267,634	185,583	167,393	167,393
A.12.PC109	141,430	98,218	90,621	90,621
A.15.PC109	31,371	17,137	14,521	14,165
A.18.PC109	187,375	127,442	118,961	118,961
A.00.PC080	92,307	64,423	50,202	50,171
A.01.PC080	19,485	9677	6150	5608
A.02.PC080	833,597	662,201	642,565	559,563
A.03.PC080	148,514	101,921	83,858	83,858
A.06.PC080	124,439	77,008	61,544	61,544
A.09.PC080	143,570	92,465	85,825	83,763
A.12.PC080	81,589	60,777	55,369	51,759
A.15.PC080	1,323,890	865,096	843,740	760,262
A.18.PC080	119,740	79,110	72,183	72,163
A.00.PC081	92,245	64,093	49,084	46,595
A.01.PC081	838,331	379,537	361,354	327,424
A.02.PC081	25,216	12,333	8036	8023
A.03.PC081	118,188	73,497	61,281	61,281
A.06.PC081	82,393	58,888	52,102	52,102
A.09.PC081	106,907	77,207	71,187	70,913
A.12.PC081	79,476	57,604	53,997	53,342
A.15.PC081	458,853	311,781	301,662	300,314
A.18.PC081	106,467	73,017	66,373	66,033
E.00.PC090	24,262	10,699	7219	7143
E.01.PC090	48,431	29,417	24,068	23,593
E.02.PC090	46,804	24,611	19,392	15,353
E.03.PC090	49,098	25,860	21,592	20,590
E.06.PC090	79,670	42,153	34,642	34,164
E.09.PC090	332,581	265,858	256,709	222,247
E.12.PC090	100,181	80,915	74,868	67,079
E.15.PC090	258,916	193,686	182,109	156,611
E.18.PC090	336,208	284,401	257,381	237,627
E.00.PC093	322,399	147,761	116,776	116,204
E.01.PC093	49,703	25,592	17,535	17,096
E.02.PC093	58,276	33,327	25,422	24,861
E.03.PC093	45,520	26,004	19,270	18,465
E.06.PC093	56,062	31,705	27,469	26,484
E.09.PC093	325,344	251,617	230,876	209,885
E.12.PC093	219,128	175,834	160,302	152,623
E.15.PC093	254,499	204,759	186,442	174,341
E.18.PC093	303,787	251,782	229,483	212,524
E.00.PC095	34,625	24,928	17,392	17,241
E.01.PC095	59,004	30,047	23,261	22,429
E.02.PC095	57,189	41,043	34,661	28,885
E.03.PC095	24,276	13,277	10,733	10,573
E.06.PC095	53,566	31,332	27,463	26,670
E.09.PC095	338,242	247,953	234,644	195,651

## Appendix 5

**TABLE A3:** Summary of SIMPER analyses showing the five taxa that most contributed to compositional dissimilarities between sampling areas (A, C and E) and sediment layers relative to the Sulfate‐Methane Transition Zone (SMTZ), for the ANME‐SRB consortium and the remaining prokaryotic community. Taxa are ordered by decreasing average contribution to Bray–Curtis dissimilarity.

Community	Contrast	Top contributing taxa (ranked)
*ANME‐SRB*	Area (A vs. E)	Methermicoccaceae; *Desulfoglaeba*; Desulfarculales; *Desulfatiglans*; *ANME‐1a*
*ANME‐SRB*	Area (A vs. C)	Methermicoccaceae; *Desulfoglaeba*; Desulfarculales; *Desulfatiglans*; Desulfobulbales
*ANME‐SRB*	Area (C vs. E)	Methermicoccaceae; Desulfarculales; *Desulfatiglans*; Desulfobulbales; *Desulfoglaeba*
*ANME‐SRB*	SMTZ (within vs. below)	Methermicoccaceae; *Desulfoglaeba*; Desulfarculales; *Desulfatiglans*; *ANME‐1a*
*Other*	Area (A vs. E)	JS1; *Sulfophobococcus*; *Methyloceanibacter*; *SCGCAAA011‐D5*; Lokiarchaeia
*Other*	Area (A vs. C)	JS1; *Sulfophobococcus*; *Methyloceanibacter*; Aerophobales; *SCGCAAA011‐D5*
*Other*	Area (C vs. E)	JS1; *Sulfophobococcus*; *Methyloceanibacter*; Aerophobales; Lokiarchaeia
*Other*	SMTZ (within vs. below)	JS1; *Sulfophobococcus*; Aerophobales; *SCGCAAA011‐D5*; Lokiarchaeia
